# Population genomics of *Aedes albopictus* across remote Pacific islands for genetic biocontrol considerations

**DOI:** 10.1371/journal.pntd.0013414

**Published:** 2025-08-11

**Authors:** Sangwoo Seok, Adam E. Vorsino, Travis C. Collier, Limb K. Hapairai, Christopher M. Jacobsen, Jeomhee M. Hasty, Ana L. Romero-Weaver, Eva A. Buckner, Dennis A. LaPointe, Mark K. H. Leong, Leo Braack, Christine A. Tabuloc, Joanna C. Chiu, Robyn Raban, Omar S. Akbari, Yoosook Lee

**Affiliations:** 1 Florida Medical Entomology Laboratory, Department of Entomology and Nematology, Institute of Food and Agricultural Sciences, University of Florida, Vero Beach, Florida, United States of America; 2 Strategic Habitat Conservation Program, Ecological Services, Pacific Islands Fish and Wildlife Office, United States of America Fish and Wildlife Service, Honolulu, Hawaii, United States of America; 3 Independent Researcher, Vero Beach, Florida, United States of America; 4 Pacific Island Health Officers’ Association, Honolulu, Hawaii, United States of America; 5 Vector Control Branch, Hawaii State Department of Health, Hilo, Hawaii, United States of America; 6 United States of America Geological Survey, Pacific Island Ecosystems Research Center, Honolulu, Hawaii, United States of America; 7 Environmental Health, Department of Public Health, Tripler Army Medical Center, Honolulu, Hawaii, United States of America; 8 Malaria Consortium, Faculty of Tropical Medicine, Mahidol University, Bangkok, Thailand; 9 Department of Entomology and Nematology, College of Agricultural and Environmental Sciences, University of California, Davis, California, United States of America; 10 School of Biological Sciences, Department of Cell and Developmental Biology, University of California, San Diego, California, United States of America; QIMR: QIMR Berghofer Medical Research Institute, AUSTRALIA

## Abstract

Remote Pacific islands (RPI) are characterized by ecological isolation, diverse endemic species, and vulnerability to invasive organisms due to globalization-driven connectivity. Among these species, *Aedes albopictus*, a highly invasive vector of flaviviruses, has spread extensively across the RPI via human-mediated dispersal, posing significant health and economic burdens. While the population structure and the degree of gene flow between mosquito populations can inform the dispersal pathways critical for disease vector management, the population genetics of *Ae. albopictus* in Northern RPI remains understudied. The present work investigated the population structure and connectivity of *Ae. albopictus* populations from Guam, Hawaiian Islands, and the Republic of the Marshall Islands (RMI) to inform disease and vector-based biosecurity risks and develop targeted management strategies. This is the first assessment to develop and analyze whole genome sequences of *Ae. albopictus* for RPI, enabling more accurate estimates of differentiation, admixture, and ancestry. We found distinct genetic clustering between regions, distinct ancestry of populations across RPI, and potential invasions that originated from Hawaii and spread into the RMI, and invasions from North America that spread to Guam. These findings can inform biosecurity protocols to limit the invasion of *Ae. albopictus* and their associated diseases within Hawaii and around the Pacific. Given the significant degree of genetic differentiation, we found between islets, islands, and regions, the genome data from this study can be used to enable the development of locally confined geographically isolated gene drives. These drives may be used to prevent and control outbreaks of dengue, chikungunya, and Zika, diseases that have had devastating consequences in these remote island communities.

## Author summary

Remote Pacific islands are home to many indigenous species, but they are also vulnerable to invasive species due to global travel and trade. One of the invasive species, *Aedes albopictus*, is the Asian tiger mosquito known to spread pathogens like dengue, chikungunya, and Zika viruses. This region has experienced increased mosquito-borne viral disease outbreaks in the past decade. In this study, we examined the genetic relatedness of *Aedes albopictus* mosquitoes collected from Guam, Hawaiian Islands, and the Republic of the Marshall Islands, along with samples from America and Asia. Our results showed that *Aedes albopictus* in each region have distinct genetic signature, and the Republic of the Marshall Islands and Guam populations likely came from Hawaii and North America, respectively. This finding helps to understand how this mosquito spreads across the Pacific region, which can improve biosecurity strategies. Furthermore, genetic information of each island population could contribute to the development of biogenetic mosquito control programs, helping to prevent future outbreaks in the Remote Pacific islands.

## Introduction

The remote Pacific islands (RPI) region supports thriving indigenous communities and diasporas, developing economies, and hosts military bases of regional and global powers [1,2]. This region is characterized by its remoteness in relation to much larger landmasses (> 350 km) [3–6]. These island systems lend themselves to invasion by non-native organisms due to complementary biotic and abiotic resource needs and ecological naiveté [7]. The connectivity of this region with larger economies via tourism, global trade, and defense posturing has resulted in the inadvertent introduction of several non-native species, including the Asian tiger mosquito (*Aedes albopictus*) [8], which have catalyzed the introduction of pathogens and disease outbreaks. Additionally, outbreaks of arboviruses such as dengue, chikungunya, and Zika, have had devastating consequences on these remote island communities [8]. Due to the concomitant invasion by competent non-native culicid vectors, as well as the presence of competent endemic culicid vectors, these outbreaks are a health and economic burden exacerbated by RPI isolation [9,10].

*Aedes albopictus*, a public concern not only in the RPI, but also globally, is thought to have originated in Southeast Asian forests [11–13]. The global expansion of *Ae. albopictus* was first documented in Hawaii in the early 1900’s [14]. *Aedes albopictus* arrived in Hawaii 5–15 years after the introduction of *Ae. aegypti*, but soon replaced it, reducing the proportion of *Ae. aegypti* to undetectable by 1949. The presence of *Ae. albopictus* on Guam was first observed in March 1948 [15], although its introduction likely predates the observation [16]. On Guam, *Ae. albopictus* larvae have been shown to outcompete *Ae. guamensis*, which is an indigenous species in Guam, but has not replaced it [17]. In 2019, dengue fever occurred in Guam for the first time in 75 years, and *Ae. albopictus* is suspected to be the primary vector responsible for this local transmission [18]. The first report of *Ae. albopictus* in the Republic of the Marshall Islands (RMI) was in 1981 [19]. In the RMI, *Ae. albopictus* is now found in rural and urban areas where it plays a major role as a vector for dengue, chikungunya, and Zika [20,21].

As the RPI is a vast expanse with comparatively little land area, invasion by *Ae. albopictus* across the expanse is characterized by long-range inadvertent dispersal through the movement of goods and people [22]. Population connectivity analyses of *Ae. albopictus* may help characterize the movement of advantageous alleles such as insecticide resistance and mosquito-borne diseases between islands. Direct assessment of genetic differentiation between sites can help identify arboviral disease and vector-based biosecurity risks and focus management actions. For controlling invasive populations on an island, one major risk is the potential for the target species to recolonize neighboring islands. Assessing population structure and connectivity using population genetic analysis is useful in understanding biosecurity risks associated with unintentional movement of this species. These types of analysis are foundational to the integrated pest management (IPM) of vector-borne diseases [23,24]. In addition, this approach can identify the causes of control failure by comparing emerging invasive populations with populations targeted during the control efforts [25,26]. While the spread of *Ae. albopictus* from its natal Southeast Asian to invasive distribution in the Americas and the Pacific has been well characterized [22,27,28], scant research effort has been devoted to characterizing the population structure and movement of *Ae. albopictus* in the RPI. In this study, we focused on the population structure of *Ae. albopictus* in three relatively overlooked populations in RPI: Guam, Hawaii, and RMI.

Although *Ae. albopictus* first started to spread in Oceania in the 19th century, its invasion of the American, African, and European continents appears to begin in the mid-to late-20th century [14,29,30]. This human-mediated global invasion of *Ae. albopictus* seems to be divided into two clusters that are primarily associated with ecophysiological traits: cold-tolerant eggs and photoperiodic diapause [29]. Individuals that invaded colder regions were derived from native cold-tolerant Asian populations, while those that invaded warmer climates came from areas where photoperiodic diapause and cold-tolerant eggs were unnecessary [23,27,31,32]. This can be also interpreted as the invasion of *Ae. albopictus* populations with different phenotypes can occur anywhere but the ones that happen to survive well and establish to a particular region had specific phenotypes that are advantageous to the invaded region. There appears, however, to have less than anticipated genetic differentiation in the invasive range of *Ae. albopictus* as compared to its native range, except for populations in Hawaii, which were established more than 100 years ago [14,29,31].

Nuclear and mitochondrial markers appear to show conflicting information regarding the genetic relatedness of *Ae. albopictus* populations. Global analyses of the genomic DNA from native and invasive populations indicated strong connections between distant geographic regions [31]. In contrast, analyses of maternally inherited mitochondrial DNA identified five major haplogroups that characterized the native range of *Ae. albopictus*. Of those mitogenome haplogroups identified by [27], only one, mitogenome haplogroup A1, was associated with the worldwide spread of *Ae. albopictus*. In other mosquito species, a discrepancy between mitochondrial and nuclear gene genealogy has also been reported [33,34]. Therefore, it is important to analyze the genetic relationship of these two different markers (nuclear and mitochondrial) to better understand the evolutionary history of *Ae. albopictus*.

Genetic biocontrol methods such as localized gene-drives are geographically or behaviorally restricted and are limited in application to a targeted, locally specific population [35]. These genetic mechanisms use either threshold dependence or locally fixed alleles to target a geographically or genetically distinct group [36,37], which effectively reduces the likelihood of non-target interactions, or other unintentional risks, associated with other broad spectrum, chemical, genetic, or even classical biological control techniques [36–38]. The development and selection (e.g., either threshold-dependent or locally fixed allele methods) of a localized gene drive are dependent on the genetic differentiation of the targeted population in relation to all others within the region of interest, as well as the ecology of the area. Therefore, an accurate assessment of population structure is needed prior to both development and application. Additionally, the effective development of a locally fixed allele-type gene drive requires an accurate genetic profile of the targeted population in order to select the appropriate genetic construct and effectively develop the approach [35].

Here, we employ a whole genome sequencing analysis to assess the population structure of *Ae. albopictus* populations in the RPI. These sequences can reveal genetic relationships and can inform localized gene drive development for spatially confined and species-specific genetic biocontrol for the RPI. We used both mitogenome and nuclear genome data from these populations to compare genetic relationships among *Ae. albopictus* populations. We hypothesized that there was significant genetic differentiation within and between islands. The connectivity of *Ae. albopictus* between islands is assumed to be driven by the movement of commodities such as used tires, potted plants, and containers. We hypothesized that the populations that were more recently established would have the lowest nucleotide diversity and the relative nucleotide diversity difference may inform the direction of mosquito dispersal within our study regions. Characterizing the genetic connectivity of mosquito populations can therefore illuminate the specific trade routes and items facilitating their dispersal. This understanding is crucial for identifying areas of arboviral disease and vector-based biosecurity risk, which directly impact disease and vector control efforts [39]. Ultimately, the analysis of *Ae. albopictus* genetic connectivity, in conjunction with trade route and commodity identification, is expected to inform and improve current and future vector management strategies [40]. This foundational assessment can further enhance management as individual collections become available from ports of entry.

## Methods

### Sampling

**Fig 1:**
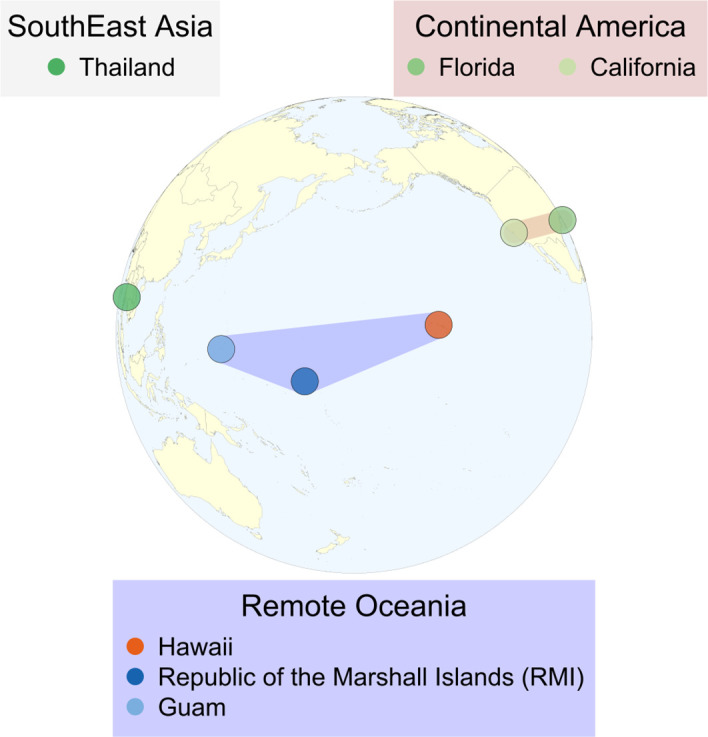
Populations included in this study. Continental USA (Florida, California), Southeast Asia (Thailand), and the Pacific Islands (Guam, Hawai‘i, and the Republic of the Marshall Islands). The ortho map was developed in the R statistical environment using the wrld_simpl dataset (https://github.com/nasa/World-Wind-Java/tree/master/WorldWind/testData/shapefiles).

A total of 133 specimens from three countries (the United States of America (USA; continental USA, Hawaii, and Guam), the Republic of the Marshall Islands (RMI), and Thailand, representing 43 *Ae. albopictus* populations were collected between 2015 and 2022 (Table 1). Samples were collected using various methods such as larval dipping, BG sentinel traps (Biogents AG, Regensburg, Germany), sweeping, and oviposition cups, and then stored in 70% ethanol upon collection before DNA extraction. For larval samples, individuals were collected from multiple habitats, with no more than two individuals collected from the same site to minimize the possibility of kin sampling. The Hawaiian Islands populations came from four different islands: Kaua‘i, O‘ahu, Maui, and the Island of Hawai‘i. Though our analysis focuses on the RPI (i.e., specimens from Hawaii, RMI, and Guam), collections from North America (California and Florida) and Asia (Thailand) were included to allow comparison of regional population structure and to evaluate invasion history. The RMI samples were collected from the U.S. Army Garrison Kwajalein Atoll leased islands; Kwajalein, Ennylabegan, and Gellinam. Fig 1 shows the collection locations for all specimens used in the analysis. The detailed sample information is provided in S1 Table.

**Table 1:** *Aedes albopictus* sample and sequencing information.

Location	Number of samples	Years collected	Av. % Mapped reads	Av. Coverage	Av. Median insert sizes
Hawaii-Kaua‘i (HK)	8	2020	93.32	13.21	252.6
Hawaii-O‘ahu (HO)	16	2022, 2023	95.59	17.14	330
Hawaii-Maui (HM)	13	2022	95.73	10.09	324.7
Island of Hawai‘i (IH)	56	2019, 2021, 2022	95.90	15.06	298.2
Republic of the Marshall Islands (RMI)	16	2015, 2018, 2021	96.06	19.33	293.5
Guam (GU)	4	2020	94.67	15.05	412.3
Florida (FL)	14	2021	95.00	16.23	318.4
California (CA)	3	2017	94.70	10.29	429
Thailand (TH)	3	2021-2022	95.10	11.36	301
Total	133	2015, 2017-2022	95.57	15.16	309.9

### DNA extraction and high-throughput sequencing

Genomic DNA was extracted using the protocol described [41]. DNA concentrations were measured using the Qubit dsDNA HS Assay Kit (Life Technologies, Carlsbad, CA) for each sample. A genomic DNA library was constructed with the QIAseq FX DNA Library UDI kit (Qiagen, Valencia, CA) using 20 ng input DNA for each mosquito. Enzymatic fragmentation was carried out at 32°C for 10 minutes, followed by end-repair and A-tailing step at 65°C for 30 minutes. Ligation of adapters was performed at 20°C for 2 hours. Library cleanup was conducted using PCRClean DX (Aline Biosciences, Woburn, MA). After post-ligation cleanup, PCR amplification of constructed libraries was conducted for eight cycles of denaturation at 98°C for 20 seconds, annealing at 60°C for 30 seconds, and DNA extension at 72°C for 1 minute. After post-amplification cleanup, library concentrations were measured with a Qubit dsDNA HS Assay Kit (Life Technologies, Carlsbad, CA) and a Qubit instrument (Life Technologies, Carlsbad, CA). The DNA library was sequenced for 150 bp paired-end reads using a NovaSeq 6000 or NovaSeq X instrument (Illumina, San Diego, CA) at the University of Florida Interdisciplinary Center for Biotechnology Research.

### Sequencing data cleanup processing and assembly

Raw sequencing reads were trimmed using *fastp* ver. 0.20.1 [42]. Trimmed reads were mapped to an *Ae. albopictus* mitogenome (NC_006817) using *BWA-MEM* ver. 0.7.15 [43] to filter out the mitochondrial reads that may be spuriously mapped to the nuclear genome, as documented in *Ae. aegypti* [44]. The mitochondrial reference genome was used in combination with *Novoplasty* ver. 4.2 [45] to construct *de novo* mitogenome assemblies from each library. We established a consistent starting position for the circular mitogenome and ensured that the orientation was aligned with published mitogenomes. A Basic Local Alignment Tool (BLAST; National Center for Biotechnology Information) search of the mitogenome was also conducted to confirm the species identity of those samples which had a mapping percentage to AalbF5 less than 95%. After mitogenome mapping, the remaining unmapped reads and ‘mate-is-unmapped’ reads were mapped to the AalbF5 reference genome (GCA_035046485.1) using B*WA-MEM* [43]. Joint variant calling using all *Ae. albopictus* samples was conducted using *Freebayes* ver. 1.3.6 [46]. In accordance with the recommendations of [47], we employed a minimum depth of eight to call variants for each individual and set a threshold of 10% maximum missing data per single nucleotide polymorphism (SNP) for filtering.

### Data analysis

To minimize the effect of differences in sample size between populations, we calculated Hudson’s F_ST_ estimates with 1,000 bootstraps for genetic distance [48]. The estimate ranges from 0 to 1, with a value of 0 illustrating no differentiation, and a value of 1 showing complete differentiation between the compared populations. Then, a Mantel test was conducted to assess the relationship between geographical distance and genetic differentiation among populations with 999 permutations [49]. The geographical location of each population was represented by GPS coordinates of the centroid of each region. We measured great-circle distance to reflect the actual physical distance between populations. With Hudson’s F_ST_, the variable represents the total differentiation in relation to the population, not the sample. Nucleotide diversity (π) was calculated across the three chromosomes using 1 Mb non-overlapping windows.

A principal component analysis (PCA), a non-parametric multidimensional technique, was used to explore the relationship between *Ae. albopictus* collected across the RPI. Additionally, multiple admixture analyses, assessments of relationships based on maximum likelihood, were performed using *Admixture* ver. 1.3.0 [50] to evaluate the heterogeneity of population structure and individual genetic ancestry across the RPI. Prior to conducting any multivariate analyses, SNP data were cleaned and filtered using *VCFtools* ver. 0.1.16 [51] and *PLINK* ver. 1.90b6.21 [52] to ensure data compatibility. Sequences were filtered by removing indels, retaining biallelic variants, and setting the minimum depth to 8 and the maximum depth to 120. Half-calls were treated as missing data, and variants with 10% or more half-calls were removed. Linkage disequilibrium-based SNP pruning was performed using a sliding window size of 50 SNPs, a window shift of five SNPs, and a Variance Inflation Factor threshold of 0.2. Three individual *Admixture* runs were conducted, varying only over the hypothesized ancestral population value (*K*) of two through four. A 10-fold cross-validation procedure was applied to each run, allowing for comparison of predictive accuracy and determination of the optimal K value.

Comparing nuclear to mitochondrial genomes is of interest to this analysis in that these two genomes, though within the same cell, have different evolutionary histories and inheritance patterns. Analyzing and comparing both genomes to each other allows for a more comprehensive and robust assessment of evolution [53]. Phylogenomic analysis via whole genome phylogenetic tree construction shows the similarity of the selected samples using branch length, and gives an indication of population evolution and ancestry [54,55]. For this analysis, the complete mitochondrial (mitogenome) and genomic DNA (nuclear genome) were analyzed separately to compare the differential evolutionary rates and population origins between them. For the mitogenome, phylogenetic tree construction was conducted using maximum likelihood (ML) to assess its predictive accuracy [56]. Mitogenome sequences were aligned using the MUSCLE algorithm in the R package *msa* [57]. The ML tree was generated using the *phangorn* package of the R statistical environment [58,59]. In *phangorn,* the best performing nucleotide substitution model was assessed using the MODELTEST algorithm, the ML distances were defined, and 1,000 bootstrap replicates were conducted to assess the robustness of the phylogenetic inference.

The nuclear genome size combined with the computational demands and memory used by the ML algorithm was excessively large even for the high-performance U. S. Geological Survey (USGS) Hovenweep Supercomputer employed for the nuclear genome phylogenetic analysis, therefore the neighbor-joining (NJ) method was applied [60]. Following the methods outlined in [55], we first identified informative sites using the methods outlined above, stored them as a variant call format (VCF) file, and transformed them into a GENLIGHT object using the package *adegenet*. Using the *popR* package, we then ran the NJ analysis for 500 bootstrap replicates to assess the robustness of the phylogenetic inference and visualize the tree [61,62].

The population phylogenies developed here were used to compare the population ancestry derived from the nuclear genome NJ tree to those inferred from the mitogenome ML tree. The tanglegram used to compare phylogenies was generated using the *dendextend* and *phytools* packages [63,64].

These analyses were conducted in either a Python environment, R statistical environment, or Linux terminal accessing specific software. Processing and assembling sequences were conducted in a custom-built workstation with AMD Ryzen 9 3950X 16 core CPU 128Gb memory. The Calculations of the F_ST_ values, PCA, and admixture analysis were performed on a Dell OptiPlex 7040 with Intel Core i7-6700. Generating phylogenetic trees was conducted on a Dell Precision 7870 intel Core i9 or the United States Geological Survey (USGS) supercomputer Hovenweep [60].

## Results

We identified 10,106,876 SNPs in our 133 *Ae. albopictus* genome dataset, which encompasses Thailand, continental USA, and RPI. The sequences for both the mitochondrial DNA (mtDNA) and nuclear DNA (nDNA) used in this study are stored in the Genbank repository. The Genbank identification numbers and SRA accession numbers are provided in S1 Table.

### Genetic differentiation and diversity

**Fig 2:**
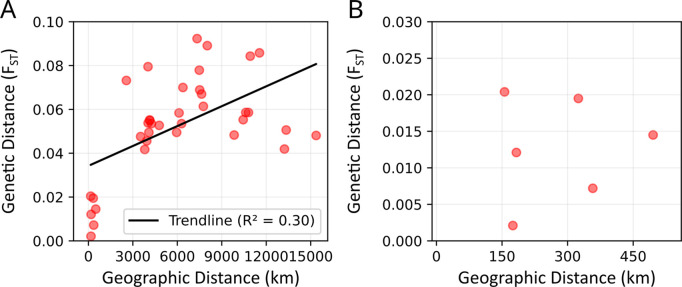
Results of the Mantel test for A) all population pairs (p = 0.004) and B) Hawaiian population pairs (p = 0.833). The X-axis represents the geographical distance between two populations, calculated as the great-circle distance, and the Y-axis represents the genetic distance (F_ST_) between the two populations.

Four Hawaiian populations (HK, HO, HM, and IH) showed very low F_ST_ values, indicating high genetic similarities among populations within the Hawaiian archipelago (or state) (Table 2). RMI displayed relatively higher F_ST_ values across comparisons, with values ranging from 0.0417 (HK) to 0.0923 (TH), reflecting substantial genetic differentiation from both Pacific (Hawaii and Guam (GU)) and continental populations. GU showed closer genetic similarity to continental U.S. populations than to RMI. The results of the Mantel test indicated a positive correlation between geographical distance and genetic distance among the entire *Ae. albopictus* populations (r = 0.55, p = 0.004), while the correlation between geographic and genetic distance among Hawaiian populations was not significant (r = 0.10, p = 0.833) (Fig 2).

**Table 2:** F_ST_ value between populations. HK = Hawaii-Kaua‘i, HO = Hawaii-O‘ahu, HM = Hawaii-Maui, IH = Island of Hawaii, RMI = Republic of the Marshall Islands, GU = Guam, FL = Florida, CA = California, and TH = Thailand.

	HK	HO	HM	IH	RMI	GU	FL	CA	TH
**HK**		0.0021	0.0072	0.0145	0.0417	0.0495	0.0614	0.0535	0.0553
**HO**	0.0021		0.0121	0.0195	0.0456	0.0584	0.0670	0.0549	0.0586
**HM**	0.0072	0.0121		0.0204	0.0495	0.0535	0.0689	0.0538	0.0586
**IH**	0.0145	0.0195	0.0204		0.0552	0.0700	0.0779	0.0795	0.0843
**RMI**	0.0417	0.0456	0.0495	0.0552		0.0732	0.0858	0.0891	0.0923
**GU**	0.0495	0.0584	0.0535	0.0700	0.0732		0.0419	0.0483	0.0527
**FL**	0.0614	0.0670	0.0689	0.0779	0.0858	0.0419		0.0476	0.0481
**CA**	0.0535	0.0549	0.0538	0.0795	0.0891	0.0483	0.0476		0.0506
**TH**	0.0553	0.0586	0.0586	0.0843	0.0923	0.0527	0.0481	0.0506	

**Fig 3:**
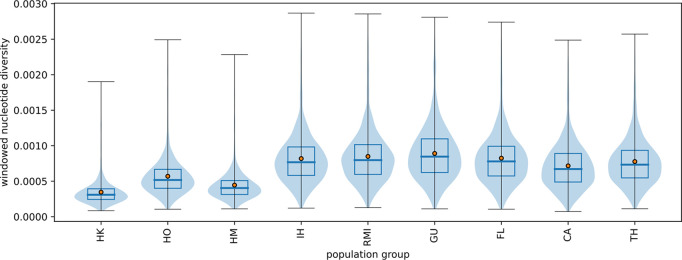
Windowed nucleotide diversity for groups of islands and regions. Violin plots represent the density distribution, and boxplots indicate the 25th, 50th (median), and 75th percentile. Red dots indicate the average value. HK = Kaua‘i (N = 8), HO = O‘ahu (N = 16), HM = Maui (N = 13), IH = Island of Hawai‘i (N = 56), RMI = Republic of the Marshall Islands (N = 16), GU = Guam (N = 4), FL = Florida (N = 14), CA = California (N = 3), and TH = Thailand (N = 3).

There is a notable variation in nucleotide diversity among Hawaiian groups (Fig 3). Island of Hawai‘i (IH) specimens had the highest nucleotide diversity, followed by HO. The Island of Hawai‘i (IH) and other non-Hawaiian regions (RMI, GU, FL, CA, and TH) showed comparable nucleotide diversity levels.

### Population structure analysis

**Fig 4:**
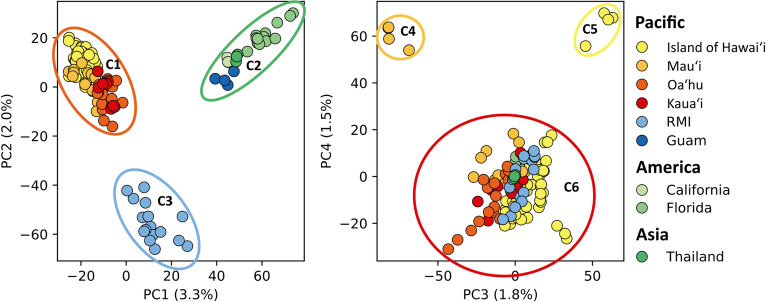
Principal Component Analysis (PCA). The left panel has PC1 and PC2 axes, while the right panel has PC3 and PC4 axes, with the percentage of variance explained in parentheses. Colors represent different geographic regions.

Principal Component 1 (PC1) and PC2 together explained 5.3% of the variance, which separated *Ae. albopictus* populations into three distinct clusters (Fig 4). These three clusters can be identified as the Hawaiian cluster (C1), the America-Asia-Guam cluster (C2), and the RMI cluster (C3). PC3 and PC4 accounted for 3.3% of the total variance and highlighted the separation of some individuals from Maui and the Island of Hawai‘i from other individuals, even within the same populations along PC4. Four samples from Kahului Airport, Maui (C4), and four samples from Puʻuhonua o Hōnaunau National Historical Park, Island of Hawai‘i (C5), were distinct from other individuals (C6). Except for C4 and C5 samples, the majority of Hawaiian individuals (92.47%) collected from four Hawaiian Islands belong to the C6.

**Fig 5:**
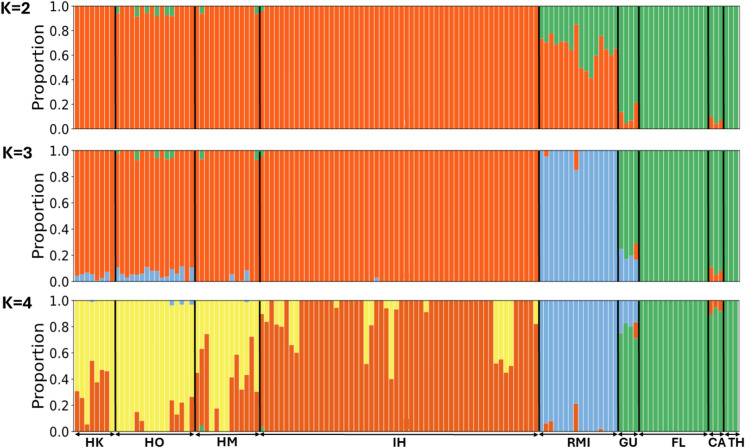
Admixture results with K = 2-4. Each horizontal panel shows the Admixture results obtained with different K values, where K is the number of genetic ancestral populations. The X-axis indicates different population groups, while the Y-axis represents the proportion of ancestry, ranging from 0 to 1. Each vertical bar represents an individual. The proportion of each color in a vertical bar reflects the estimated ancestry fraction from each ancestry population. HK = Kaua‘i, HO = O‘ahu, HM = Maui, IH = Island of Hawai‘i, RMI = Republic of the Marshall Islands, GU = Guam, FL = Florida, CA = California, and TH = Thailand.

Bayesian clustering analysis using Admixture revealed distinct genetic structures among populations. The cross-validation (CV) error value for our Admixture run was the lowest at K = 2 (S1 Fig). This indicates that the data is consistent with two ancestral populations of *Ae. albopictus* in our dataset with the RMI population likely an admixture of the two ancestral populations. However, given our PCA results, K = 2 solution may not be the most optimal solution to succinctly explain the population structure among *Ae. albopictus* populations. The entropy values provide the informativeness or uncertainty in the population assignment and can be used to find the minimum number of clusters explaining the data with the minimum entropy [65]. Using the entropy measures, K = 3 appears to be the most succinct clustering. Nonetheless, the order of subdivision in Admixture run can be informative in identifying the genetic clusters with largest differentiation (K = 2) and subsequent clusters with lesser genetic differentiation (K = 3 or higher). Therefore, we provided K values between 2 and 4 (Fig 5). At K = 2, when two main ancestral groups were used, Hawaiian populations were associated with one group, while individuals from Guam and other continental populations (FL, CA, and TH) exhibited another ancestral group (Fig 5). The RMI population appeared to have a mixed group of these two ancestral groups. When a third ancestral group was added (K = 3), the distinctiveness of the RMI became evident. This result is consistent with PCA results with RMI individuals clustering separately from the other two clusters. At K = 4, the genetic composition of the Hawaiian populations (HK, HO, HM, IH) varied by island. Populations from HO and IH showed a high proportion of a single ancestral group, yet these dominant groups differed between the two islands. In contrast, individuals from HK and HM exhibited a blend of two ancestral groups, which correspond to the ancestral groups found on HO and IH. The RMI population maintained a unique ancestral profile. Conversely, the GU population continued to share the same ancestral group with continental U.S. populations.

### Phylogenetic analysis

**Fig 6:**
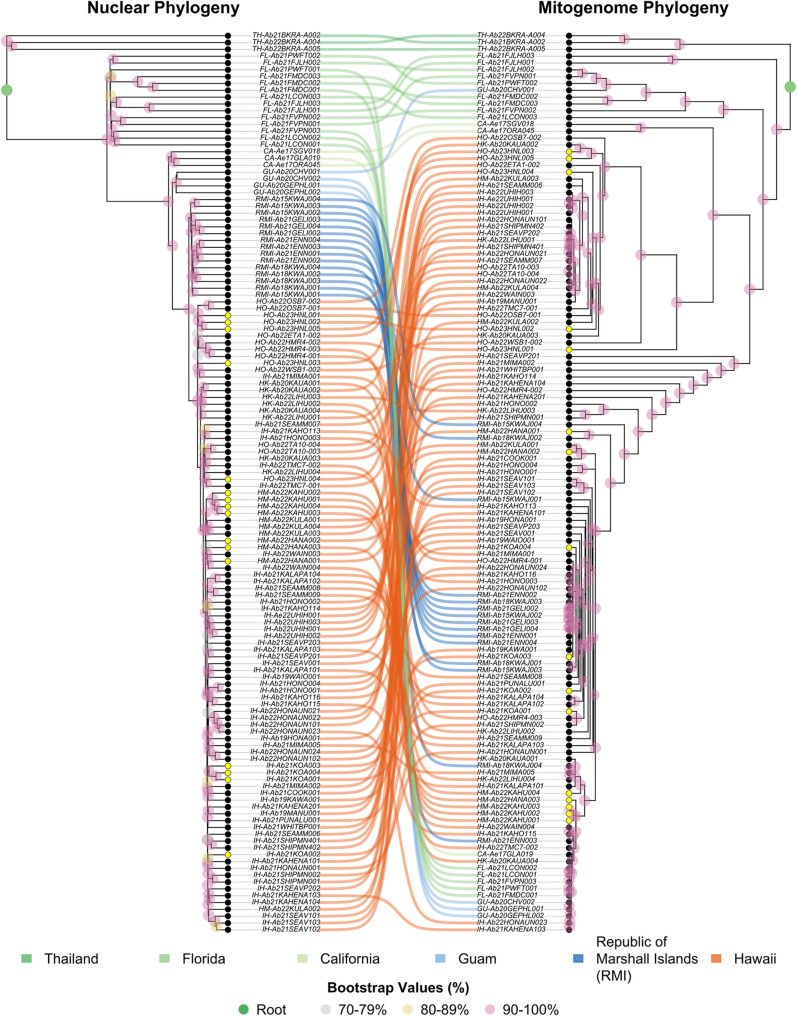
Phylogeny for *Ae. albopictus* nuclear (left) and mitochondrial genomes (right) with bootstrap values and linkage of the samples between the two phylogenies. The color of the sample linkage between the sample genomes corresponds to the primary location of the collection. Bootstrap value percentages >70% are shown, with colors representing increments of ten percentage points. Both phylogenies are rooted by a natal population of *Ae. albopictus* from Thailand. The nuclear phylogeny was developed using the neighbor-joining method (500 bootstrap replicates), while the mitochondrial phylogeny was developed using the maximum likelihood method (1000 bootstrap replicates). Tips colored yellow are associated with collections from ports of entry (e.g., airports). The substitution model TVM + G(4)+I was used to develop the mitochondrial maximum likelihood phylogeny. Island names consistent with Table 1 are given next to sample name id.

The nuclear genome tree identified discrete differentiation and ancestry when comparing geographically defined populations, with partitioning similar to the Admixture analysis (Fig 6). When compared to the samples from TH, the FL samples were ancestral to all other samples, likely indicating a more recent invasion from the Asian origin of *Ae. albopictus*. The samples from CA were partitioned separately from those collected in FL, which interestingly were more closely related and ancestral to samples from GU. The RMI and Hawaii (HK, HO, HM, IH) sample genomes were distinctly partitioned and showed a large degree of differentiation among populations on islands.

Of the 92 substitution models tested for the mitogenome ML tree, the transversion model with a gamma-distributed rate variation that allows for invariable sites (TVM + G(4)+I) was the best performing (log-likelihood: 39250.80; BIC: 81161.20; AIC: 67569.74) (Fig 6). The mitogenome phylogenetic tree was equivalent to the nuclear phylogenetic tree in many ways, while also showing distinct patterns that could also be associated with ancestral female population invasions. For instance, samples from GU and Hawaii were co-located with those from CA, FL, and RMI, potentially indicating multiple invasions from Hawaii to RMI and maybe CA and FL. The co-location of samples from GU with FL in the mitogenome phylogeny may be indicative of higher relatedness than to Hawaii populations and the potential of multiple invasions from distinct North American regions into Guam. Though the nuclear phylogeny identified discrete population-based differentiation that in theory is associated with temporal population ancestry, the mitogenome reveals a likely more intuitive assessment of invasion history given *Ae. albopictus* invasion dates. This is especially of interest because in the United States, Hawaii (samples labeled HK, HO, HM, IH) has a much longer history of occupation by *Ae. albopictus* than those associated with the North American continent (samples labeled CA, FL, GA) or other Pacific islands (RMI, GU).

Discordance between nuclear and mitogenome phylogenies reveals an interesting relationship. In the RMI, this discordance is characterized by the distinct partitioning of the RMI samples in the nuclear phylogeny via historic introgression and sex-biased dispersal. Whereas the relationship of the samples from Florida and Guam (FL and GU) to the samples from Hawaii (IH, HK and HM) on the periphery of the mitogenome tree may not reflect an evolutionary relationship. Instead, it may have resulted from random genetic drift induced by the higher mutation rate of the mitochondrial DNA, converging these samples.

## Discussion

Despite the widespread distribution of *Ae. albopictus* in the RPI, the populations of this significant disease vector have been poorly studied [27,31]. Although several decades have passed since the initial invasion report of this species in Guam [15] and the RMI [19], to the best of our knowledge, this is the first study to investigate the population structure in these regions. Our results highlighted that the RMI populations and the Hawaiian populations in the Pacific region are genetically distinct from other populations.

For the Hawaiian populations, *Ae. albopictus* individuals from four different islands were included, and very low levels of genetic differentiation were observed. This suggests that *Ae. albopictus* populations in the Hawaiian Islands likely shared a common genetic source, and migration is not necessarily limited within and between the islands. In the PCA, a few individuals from Maui (C4) and the Island of Hawai‘i (C5) were clustered outside of the main Hawaii groupings (C6) (Fig 4). These samples belong to specific populations on Maui and the Island of Hawai‘i, suggesting that their sampling locations might have been geographically isolated from other regions due to environmental factors or have recently experienced genetic drift. All individuals collected near Kahului airport (HM-Ab22KAHU001, HM-Ab22KAHU002, HM-Ab22KAHU003, and HM-Ab22KAHU004) belong to the C4 group. However, considering that C4 is located with other Hawaiian samples in the PC1-PC2 axis graph and both nuclear and mitogenome phylogenetic trees, it is likely that genetic drift occurred following geographic isolation, or this group was introduced from a Hawaiian Islands population that was not included in this research. Interestingly, in the case of C5 on the Island of Hawai‘i, individuals belonging to this group (IH-Ab22HONAUN021, IH-Ab22HONAUN022, IH-Ab22HONAUN023, and IH-Ab22HONAUN101) were collected together with other individuals classified as C6 in Puʻuhonua o Hōnaunau National Historical Park. In the phylogenetic trees, C5 individuals clustered together, whereas individuals belonging to C6 in this region are scattered throughout both the nuclear and mitogenome phylogenetic trees in Fig 6. It infers that C5 experienced genetic drift during a period of isolation, and then some C6 individuals were recently reintroduced to this region from other Hawaiian regions. Future studies focused on intra-island genetic population differentiation may be valuable for inter-island assessments as they may better assess geographic isolation and port of entry population origin. This analysis attempts to be a foundational assessment from which those inferences can be made once additional island-specific collections are obtained.

Despite having the longest invasion history in Hawaii among the populations in this study, Kaua‘i, Maui, and O‘ahu populations showed lower nucleotide diversity compared to other RPIs in this study (Fig 3). This lower diversity may be due to (1) drift in the form of bottleneck and having less time to develop sufficient genetic differentiation between islands, or (2) frequent dispersal and gene flow pathways between islands of Hawaii, or (3) reduction of genetic variations caused by chemical and biological mosquito control efforts in response to several dengue outbreaks that have occurred in Hawaii [14,66].

The invasion route of *Aedes* mosquitoes is not well documented in this region; however, we do know the invasion route of a closely related species *Ae. japonicus,* first established on the Island of Hawai‘i, later found near an international airport on O‘ahu, and finally spread to Kaua‘i [67]. In addition, a new invasion of *Ae. aegypti* was detected relatively recently at the same international airport on O‘ahu [68]. Therefore, given that anthropogenic activities have been implicated in the spread of these three species, it is likely that *Ae. albopictus* was also first established on the Island of Hawai‘i and O‘ahu, where major ports of entry are located. The ports of entry on these islands, which include military bases and commercial airports, could explain why the two populations maintain higher genetic diversity than those on the other two Hawaiian Islands.

Human-mediated dispersal of *Aedes* mosquitoes has led to gene flow among populations, as indicated by the relationship between genetic and geographic distance [69,70]. Among the various dispersal pathways, maritime and air transportation play a significant role in facilitating that movement [69,71,72]. The non-significant result of the Mantel test, when including only the Hawaiian populations, suggests that they are not separated by geographic distance, but rather connected through anthropogenic activities (Fig 2). Given the archipelagic nature of Hawaii, where islands are interconnected through maritime and air transport, continuous gene flow is likely linked to the movement of goods and people across the Hawaiian Islands.

Compared to the RMI and Hawaiian populations, the Guam population showed a different pattern. As shown in Figs 4 and 5 and the nuclear genome phylogeny in Fig 6, the Guam population is genetically closer to the Asian and North American populations than other Pacific Islands. The first *Ae. albopictus* occurrence in Guam was reported in ~ 1945 [16]. Following the second battle of Guam in 1944 where the US army recaptured Guam after Japanese occupation of the island for over 2.6 years, Guam was turned into a base for allied operations which coincides with the first *Ae. albopictus* reporting. North American *Ae. albopictus* was reported to have originated from Japan in the 1980s [73,74]. The genetic similarity we observed between Guam and Florida indicates that the Guam population could have a founding population similar to Japan or other Asian countries. Since its founding, the Guam population may have diverged from its natal Asian genotype and evolved independently like the Hawaiian population.

The RMI population is more ancestral to the Hawaii population in the nuclear phylogenetic tree, while the mitogenomic tree has mixed geographic origin under the node separating the Thai population and the rest of the samples (Fig 6). This result may indicate that historic invasions of an ancestral population to Hawaii and the RMI have occurred, and followed by independent evolution. Notably, the RMI samples were collected from areas that have shipments originating from North America and Hawaii. However, their genetic differentiation from Asian genotypes remains high unlike the Guam population.

Collections from ports of entry such as those collected from Daniel K. Inouye International Airport in Honolulu (sampled labeled HNL) and other airports from Maui (labeled HANA), Kaua‘i (labeled KAHU), and the Island of Hawai‘i (labeled KOA) were associated with distinct monophyletic groupings of *Ae. albopictus* from within their geographic sites*,* especially in the nuclear phylogenetic tree (yellow tips in Fig 6). Therefore, the ports of entry individuals are likely derived from the geographic region in which they were collected. Even though the nuclear phylogenetic tree shows distinct monophyletic groups unique to specific islands, these groupings do not match the broader population boundaries we see in the PCA and Admixture analyses (Figs 4 and 5). Because these island-specific groups are not clearly separate from the main populations in the PCA and Admixture results, we can not use those analyses to figure out where individual samples originally came from if they were derived from locations in the Hawaiian islands.

These outcomes are also relatively consistent in the mitogenome for the Hawaii-based samples, but the mitogenome phylogeny may also suggest inter-island movement. For instance, the monophyletic groupings of port of entry samples (HO-Ab23HNL003, HO-Ab23HNL005) from O‘ahu, co-located with a sample (HK-Ab20KAUA002) from Kaua‘i suggests introgression of the mitochondria at ports of entry, but could also be explained by molecular convergence driven by genetic drift as there are other natal O‘ahu samples in higher level monophyletic groupings. Similarly, a number of other ports of entry samples from O‘ahu, Maui and the Island of Hawai‘i were co-located with natal populations outside of the collection island in the mitogenome phylogeny (Fig 6), suggesting sex-biased inter-island movement, but as above, molecular convergence driven by genetic drift cannot be specifically ruled out. Additional collections may help to better resolve the mitogenome phylogeny and clarify the presence of sex-biased inter-island movement patterns.

Samples from the RMI were co-located in monophyletic groups with samples from HK, HM, and IH in the mitochondrial phylogeny. Interestingly, a sample from Kwajalein island in the RMI (RMI-Ab18KWAJ002), and one from Hana airport on Maui (HM-Ab22HANA002) were phylogenetically proximate, potentially indicating that the Hawaiian population is a source population. These results seem consistent with the Admixture analysis results (Fig 5), though the mitogenome phylogeny identified some cryptic historic relationships that the nuclear genome-based Admixture analysis did not.

In both *Anopheles arabiensis* and *Ae. aegypti* linkage disequilibrium (non-random association of alleles at different loci) decays rapidly in the genome, therefore any nuclear differentiation is caused by more recent gene flow events, and mitochondrial inferred associations are more ancestral diversification events [35,75]. In congruence with these studies, assessing population connectivity via a focus on nuclear genomic influence may be preferable as they are more likely to be derived from current immigration events and, thus, more likely to be significant to management efforts and tool development. When interpreted this way, it is apparent that there is very little recent gene-flow between the different island systems of the RPI (Hawaii, RMI, and Guam), as indicated by the nuclear genomes, whereas past ancestral states indicated possible RMI origins of *Ae. albopictus* from Hawaii, and potential ancestral immigration between Guam and Florida. Given that the mutation rate of the mitochondria is greater than that of the nuclear genome, and the mitochondria are orders of magnitude smaller, random genetic drift might show a greater degree of kinship than reality.

Considering the > 120 years of divergence time, which is equivalent to over 6,000 generations since *Ae. albopictus* was first introduced into Hawaii. It has diverged significantly within and between islands, developing into distinct evolutionary units [14,29,31]. Due to this high level of differentiation, it is likely that some of the Hawaii mitogenomes have converged similarly to those from the RMI, Florida, and Guam, as seen in the periphery of the phylogenetic tree (Fig 6). However, for the RMI samples, discordance between the nuclear and mitogenome phylogenies reveals the distinct partitioning of the RMI samples in the mitochondrial phylogeny via historic introgression and sex-biased dispersal. Given that in the mitogenome phylogeny the RMI samples consistently are co-located with Hawaii samples at various degrees of evolutionary divergence from the phylogenies root, convergence via random genetic drift is unlikely. Therefore, we postulate that multiple introductions from Hawaii to the RMI have likely occurred, as well as introductions from Asia.

Historic introgression and sex-biased dispersal may also explain the relationship of the Florida and Guam (FL and GU) samples to the Hawaii (IH, HK and HM) samples located in the periphery (in relation to the root) of the mitogenome tree (Fig 6). However, the mitogenome phylogenies periphery encompasses the samples with the highest rates of evolutionary divergence. Therefore, the relationships in this peripheral area may be due more to random genetic drift converging these samples in the periphery via the mitochondrial genome’s higher mutation rate, rather than an evolutionary relationship. The inclusion of additional Florida and Guam samples may help to resolve this phylogenetic relationship.

*Aedes albopictus* was first introduced into Hawaii around the 1900s [14], Guam ~1948 [15,16], RMI in 1981 [19,76,77], Florida ~1986 [74] and California in ~2001 [78]. Given this history, it is notable that, except for California, the evolutionary history identified here directly reflects the invasion history of *Ae. albopictus* throughout North America and the Pacific, where the most temporally (and geographically) isolated population (Hawaii) is the most diverged from its natal ancestor.

Another important innovation of our approach is the use of a small but sufficient sample size to be able to carry out our analyses. Estimates of F_ST_ derived from whole genome sequence data have been shown to be accurate even with very small sample sizes (i.e., N = 2/population) [79]. This is due to the very large number of SNPs (i.e., n>>1,000 loci) used in these analyses. In our case, we used over 10 million SNPs for our analysis, well exceeding the number of markers required to compensate for the small sample size. We also used assignment methods that do not require accurate estimates of frequencies. Admixture method used here assumes the Dirichet prior for frequencies and then averages over the uncertainty inherent in estimates based on samples of limited sample size [50]. Other genome based-methods that were not utilized here but used small sample size and produced meaningful results. For example, as few as 15 low frequency alleles per study site led to accurate estimates of pairwise dispersal distance [80]. A change in the rate of admixture between two subspecies of *Mus musculus* using low-coverage sequences from 7 individuals of one subspecies and 8 of the other [81]. In a continuously distributed population, accurate estimates of dispersal distance can be obtained using as few as 100 sequenced individuals dispersed in space [82]. Genome-based estimates like PSMC require only small sample sizes (N = 1–2) depending on genomic coverage [83] and provide a way to estimate confidence intervals by determining the range of variation across individuals. In addition, evidence also supports the use of a single diploid individual genome sequence for making demographic inferences [84]. Therefore, relatively small sample sizes are sufficient for all these methods because recombination ensures that in effect each genome can be representative of the whole population.

Demographic history analysis as demonstrated in other systems [83,84] would be useful to resolve some invasion history scenarios we laid out in this paper. In particular, the pairwise and multiple sequentially Markovian coalescent method has been used in *Anopheles* mosquito systems [85,86]. However, this analysis requires several assumptions including (1) point mutation rate, (2) number of generations per year, and (3) genetic linkage map that anchors genomic segments into linkage values in centimorgan for accurate estimation. We have none of these for *Ae. albopictus* at the moment. Therefore, we left these more advanced genome analyses for future studies when we can make fewer assumptions when inferring the demographic history of *Ae. albopictus* populations.

The central aim of this work is to inform local vector control strategies and enable the development of localized biocontrol systems by elucidating the genetic structure of *Ae. albopictus* in the region. In this study, we were able to provide a broad picture of population structure in the remote island systems in the Pacific. The data we generated here specific to Hawaii Island (N = 91) may provide sufficient data to detect a variant as low as 0.5%. Other island systems like RMI and Guam may need more data in order to capture robust allele frequency information that may be needed for target-site resistance potential [85,87]. Despite Hawaii’s lack of native blood-feeding mosquitos, ongoing dengue outbreaks across its islands and the wider RPIs highlight a significant public health and economic threat. A localized, reversible genetic biocontrol mechanism applied in the RPIs could therefore protect not only local economies and communities but also those connected to them, by curbing the spread of the mosquito borne diseases such as dengue [8,10,14]. This study represents a foundational assessment of the RPI populations’ genetic structure from which future regionally specific analyses can be developed.

Quantifying the degree of *Ae. albopictus* differentiation, and understanding its ancestry across the Pacific is important for both prioritizing biosecurity and Integrated Pest Management (IPM) efforts, and developing more efficient and effective tools for controlling *Aedes* and their arboviral diseases. The relationships identified by the differentiation assessments and phylogenies developed here can be used to identify origins of invasion of future collections. Genetic analysis of mosquito populations can directly inform the efficient application of biosecurity protocols. By understanding the genetic links between *Ae. albopictus* populations, we can identify specific commodity pathways associated with their movement. This allows for targeted interventions, such as focused inspections of goods traveling along established *Ae. albopictus* routes throughout the RPI, ultimately bolstering regional biosecurity. Focused biosecurity measures, and IPM efforts in regions of interest, can maximize control while minimizing the cost of further invasions. Additionally, the whole genome sequences developed for this analysis may eventually enable locally confined, geographically isolated gene drives for the Pacific islands, facilitating IPM efforts in the invaded and uninvaded regions [34]. For instance, given the island and locality-based differentiation that has developed in Hawaii (Fig 4), powerful site-specific gene drive systems could be developed for specific regions in Hawaii, or be used at ports of entry (or exit) to protect from introductions from areas with active arboviral transmission. The application of these protocols and tools may reduce the high costs associated with controlling these arboviral vectors, helping to enable the future of RPI communities.

## Supporting information

S1 Fig Cross-validation (CV) errors from Admixture analyses for K values ranging from 1 to 6. (TIF)

S1 Table The sample metadata including location, GPS coordinates, SRA accession numbers, and sequence coverage information for each specimen. (XLSX)
