# Supplementary figures and images for: Population genomics of Aedes albopictus across remote Pacific islands for genetic biocontrol considerations
Source: PLoS Negl Trop Dis. 2025 Aug 11;19(8):e0013414. doi: 10.1371/journal.pntd.0013414 (PMC12352873; doi:10.1371/journal.pntd.0013414)

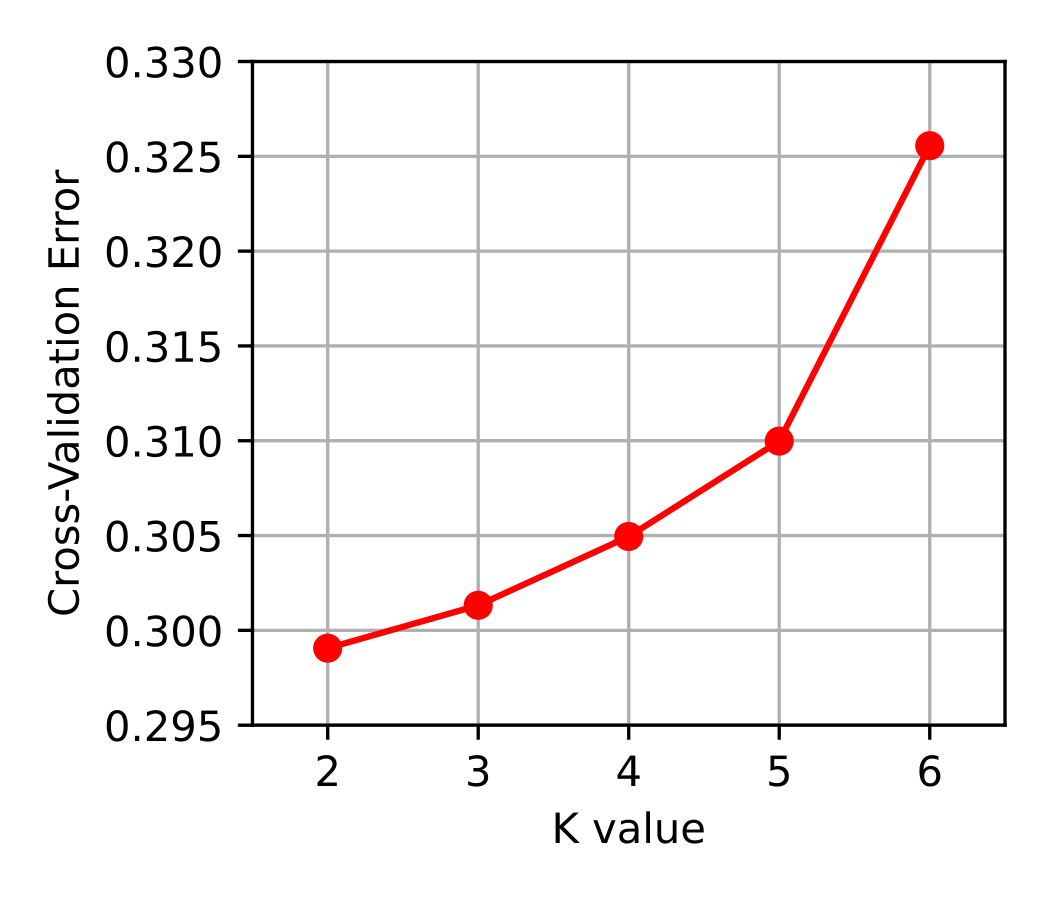

Supplement: S1 Fig — (TIF) [file pntd.0013414.s001.tif]
